# Are school-based mental health interventions for war-affected children effective and harmless?

**DOI:** 10.1186/1741-7015-12-84

**Published:** 2014-05-21

**Authors:** Verena Ertl, Frank Neuner

**Affiliations:** 1 Department of Psychology, Clinical Psychology and Psychotherapy, Bielefeld University, Post BOX: 100131, 33501 Bielefeld, Germany

**Keywords:** Trauma, Children, War, PTSD, Depression, Treatment, Prevention, Trial, Effectiveness

## Abstract

In recent years, different approaches to large-scale mental health service provision for children in war-affected, mainly low- and middle-income, countries have been developed. Some school-based programs aiming at both strengthening resilience and reducing symptoms of trauma-related distress have been evaluated. In an article published in *BMC Medicine*, Tol and colleagues integrate their findings of the efficacy of universal school-based intervention across four countries and do not recommend classroom-based intervention as a treatment of trauma-related symptoms, since no consistent positive effects were found. On the contrary, for some children this type of universal intervention may impair recovery. Since universal school-based programs similar to the one evaluated here are widely implemented, Tol *et al*.’s results are highly relevant to inform the field of mental health service provision in war-affected countries.

Please see related article http://www.biomedcentral.com/1741-7015/12/56.

## Background

Across the world, many low- and middle-income countries (LMICs) are affected by armed conflicts that threaten the healthy development of children. High rates of psychological disorders, including posttraumatic stress disorder (PTSD) and depression have been found among children living in these regions [1]. Providing mental health support for these children is challenging, since the high need for treatment is accompanied by an extremely limited availability of specialized professionals. In recent years, various attempts to meet this challenge have been made. Different prevention and intervention programs for war-affected children have been developed and adapted for use so that they can be implemented by trained paraprofessionals (such as teachers or adult members of the affected communities), some of them with promising effects in evaluation research [2,3]. The approaches vary with regard to their level of specialization (from universal to highly specialized services), the targeted ecological context (individual, family, school, community) and the timing in relation to the trauma (immediate, intermediate or long-term). Most of the current research has focused on school-based programs aimed at strengthening resilience and reducing symptoms of trauma-related distress in children with and without symptoms above a clinical threshold. School-based interventions are usually composed of modules such as psychoeducation, socio-drama, movement or dance, playful group cohesion activities, strengthening personal resources and coping as well as, in some programs, limited trauma-exposure. A prominent example of such programs is Classroom-Based Intervention (CBI), a program that aims to reduce symptoms of PTSD, depression and anxiety and to increase children’s resilience [4,5].

However, in a research article published in *BMC Medicine*, Tol and colleagues conclude in their presentation of the results of a trial and relating the findings to previous trials that CBI showed favorable as well as unfavorable effects depending on contextual and individual factors [6]. They notice that in unstable settings, the natural recovery of children that live in stressful conditions may even be undermined by the intervention. In light of this converging evidence-base the article raises an important question: Is CBI (and similar universal preventive school-based programs) safe enough for use with children in war-affected areas, or may it even be harmful for some children? Considering the limited resources for health-service provision and the potential negative effect for some children in war-affected regions, is it still justified to use universal preventive school-based programs on a large scale? Are there any alternatives? Where should research focus next?

### Research on classroom-based intervention

The recently published article by Tol *et al*. in *BMC Medicine* presents the outcomes of the last of four largely equivalent (in design, methods, and intervention) cluster-randomized-controlled trials that have been conducted by members of the nongovernmental organization HealthNET TPO, cooperating universities and implementing organizations [4-7]. All trials aimed to assess the efficacy of the prominent and widely disseminated school-based intervention CBI [8] in reducing psychiatric symptoms (depression, PTSD, anxiety) and improving sense of hope and functioning. CBI was applied to groups of war-affected children (between 7- and 17-years-old) by paraprofessionals in Indonesia, Nepal, Sri Lanka and Burundi between March 2006 and March 2008, with assessment periods ranging from post-treatment to a six months follow-up [4-7].

The most recent study presented by Tol and colleagues [6], as well as the three earlier published trials [4,5,7], demonstrate that methodologically rigorous evaluation-research can be implemented successfully in challenging contexts such as LMICs of limited political stability. Some of the common limitations in this field of research, for example, lack of randomization and small sample sizes per treatment arm, have been overcome in these studies. A major challenge for the evaluation of the efficacy of psychosocial interventions for war-affected children is the heterogeneity of treatment approaches, dosages, formats, assessments, target groups (subsyndromal versus syndromal) and follow-up periods, which makes systematic aggregation of knowledge difficult. However, since the current study by Tol *et al*. [6] in Burundi relied on the same methodology as equivalent work in Indonesia, Nepal and Sri Lanka, we can use the evidence-base from these studies to draw relatively solid conclusions about the benefits and risks of CBI across different contexts.

### Benefits and risks of CBI as treatment

With the exception of the study in Indonesia [4], the randomized trials on CBI did not show a significant treatment effect for depression, PTSD, or anxiety. The simple conclusion that can be drawn from these findings is that the widespread use of CBI is not justified, at least not for the treatment of trauma-related disorders. The group was very careful to analyze differential effects for several subgroups. However, these effects were inconsistent across studies. In Indonesia, positive effects on PTSD symptoms were found for girls, yet not for boys and no effects were found on symptoms of depression and anxiety. In Sri Lanka [7], boys and children with less ongoing trauma-exposure benefited with regard to PTSD and anxiety symptoms, yet no effects were found on symptoms of depression. In Burundi [6], children living with both parents benefited from CBI with regard to symptoms of PTSD and depression and symptoms of depression were reduced for those living in larger households. This pattern of inconsistent results has several implications. First of all, there are contextual and individual moderators that determine whether a specific child will benefit from treatment or not. These results question the use of CBI as a universal (one size fits all) intervention/prevention approach for all children. Secondly, the moderators are inconsistent across studies and all of the findings are *post-hoc*. It is, therefore, not possible for differential recommendations for the use of the intervention to be made, that is, a prediction about who will benefit from the treatment, in which context and, therefore, who should receive CBI and who should not. Thirdly, it is important to note that most trials had an overall zero effect on symptoms. The mere presence of significant moderators where there was an average zero-change is a strong indicator of the so-called deterioration effect [9]. The deterioration effect relates to the fact that the average trajectory of study participants in a trial does not necessarily inform about potential harmful effects of the intervention. It is possible, and not improbable, that some subjects benefit from a treatment while others do get worse, which may still result in an overall positive (or null) effect of the intervention. However, *primum non nocere* (first do no harm) must be the major ethical principle of any treatment, and all interventions, especially those with small overall-benefits, must show that they are not harmful for some individuals. Unfortunately, there is no information on clinically significant worsening or impaired recovery in the CBI trials, but Tol *et al*. [7] report that Sri Lankan girls with CBI were doing worse than girls in the waiting list condition in terms of their change in PTSD symptoms. Moreover, children dropping out of CBI in Burundi had significantly higher levels of PTSD symptoms [6]. The negative effect of CBI is probably not dramatic given the small or absent overall effect. However, it appears that those who suffer the most in terms of symptoms of mental health disorders benefit the least, or may even deteriorate through CBI, which questions the use of this intervention as a treatment for trauma-related disorders.

### Benefits and risks of CBI as prevention-intervention

Tol and colleagues [6] found that, while there was no effect on symptoms, CBI showed favorable results as a preventive school-based intervention in strengthening resilience. However, at the same time, the trial shows a negative development on resilience factors for the subgroup of displaced Burundian war-affected children in the intervention group. Therefore, the study indicates that universal prevention programs might interfere with the natural course of recovery and healthy readjustment of a minority of children. Tol *et al*. [6] are right to conclude that limiting the aim of CBI to a prevention program increasing resilience would possibly make the intervention more ‘safe’. A clear preventive focus may be achieved by removing the probably insufficient trauma-focused interventions from CBI, since it might not be possible to process traumatic memories with the limited individual support provided in CBI. A recent study by Ager and colleagues [10] has implemented an adapted version of CBI called Psychosocial Structured Activities (PSSA) without a trauma-component for use with war-affected children in Uganda and has found a significantly stronger increase in a local measure of child well-being in the intervention group compared to a waiting-list control. Nevertheless, without conducting dismantling studies it remains unclear whether it is really the limited trauma-component of CBI that presents a risk for some children.

## Conclusions

First of all, the findings by Tol *et al*. [6] strongly emphasize the need for research in planning, piloting and implementing both low-threshold psychosocial and highly specialized mental health interventions in LMICs. The uncritical implementation of seemingly ‘non-invasive’ and ‘safe’ interventions may, in fact, carry an unexpected risk for some participants. Moreover, it is essential that research on prevention and treatment approaches study potential negative or side effects during and after program implementation. It is tempting to perceive universal programs like CBI as a perfect solution to fill the mental health service gaps in war-affected countries, since they are low-threshold, cost-effective, resource-friendly and target many beneficiaries within a short period of time. However, the heterogeneity of beneficiaries and the lack of focus of the interventions have turned out to be problematic, as for some groups negative effects may occur. We, therefore, agree with Tol and colleagues [6] in their main conclusion that CBI, and possibly also similar interventions, should not be recommended as a treatment for symptoms of PTSD, depression and anxiety.

As an alternative, converging evidence supports a principle that is fundamentally different from the universal application of school-based programs: the screen-and-treat approach with the primacy of thorough and systematic screening of symptom severity and symptom composition. Those who suffer from clinically relevant symptoms of mental health disorders receive specialized treatment that meets their needs, while those who recover on their own do not receive unnecessary treatment. A variety of individual and group treatments have been developed for these purposes, focusing mainly on PTSD and depression, for example, Trauma-Focused Cognitive-Behavioral Therapy (TF-CBT), Narrative Exposure Therapy (NET) and Interpersonal Psychotherapy (IPT) [11-17]. The emerging evidence-base for these interventions shows that even for specialized trauma-focused psychotherapy, the employment of trained lay-therapists within a care system that provides supervision and referral is an effective alternative in the absence of clinical professionals, which makes these interventions relatively cost-effective. Within a stepped-care screen-and-treat approach there might still be space for universal preventive programs, such as a modified CBI, to increase resilience for large groups of children. However, longitudinal research is necessary to confirm that self-rated attitudes and perceptions about hope and social support, that have been the outcomes of the recent trials, translate into healthy behavior and inoculate the children against impairment and psychological problems in the long term, especially when adversities and traumatic experiences reoccur. Other school-based prevention programs aimed at strengthening resilience have been evaluated, such as ERASE-Stress [18] and Overshadowing the Threat of Terrorism (OTT) [19]. Neither has a trauma-focusing component, still they show promising results.

### Future directions

There has been a concentration on treatment for PTSD as one of the most prevalent mental health disorders, yet more research needs to include other common mental health disorders and behavioral problems that may develop following trauma. Moreover, it has been emphasized that war-experiences can foster the reoccurrence and worsening of a wide variety of pre-existing psychopathology, which requires the strengthening of a broad and functional mental health care system in addition to special trauma interventions [20]. Furthermore, there is still a need to find ways to sustainably implement specialized treatment approaches and referral structures into public health care-, school-, or NGO health care-systems in LMICs. It is difficult to identify those children who are most in need of mental health services, and to determine the best time to intervene. There is a strong decline in PTSD symptoms in the first year, if traumatic experiences do not continue. Therefore, prevention programs and early interventions - especially when resources are scarce - have to find a balance between treating those who need it and not treating those who will naturally recover or where treatment would cause harm. Concerning many of the introduced programs above, dismantling studies are needed to identify the most relevant intervention strategies. Currently, it remains unclear which of the compiled intervention strategies are having the most positive impact on which symptoms.

## Abbreviations

CBI: Classroom-Based Intervention; IPT: Interpersonal Psychotherapy; LMICs: low- and middle-income countries; NET: Narrative Exposure Therapy; OTT: Overshadowing the Threat of Terrorism; PSSA: Psychosocial Structured Activities; PTSD: posttraumatic stress disorder; TF-CBT: Trauma-Focused Cognitive-Behavioral Therapy.

## Competing interests

The authors declare that they have no competing interests.

## Authors’ contributions

VE and FN contributed to the conception of the manuscript. VE drafted the first version and both authors revised it critically for its intellectual content. VE and FN were involved in editing and revision of the manuscript, read and approved the final manuscript and agreed to its publication.
